# Symbiotic Associations in Ascidians: Relevance for Functional Innovation and Bioactive Potential

**DOI:** 10.3390/md19070370

**Published:** 2021-06-26

**Authors:** Ana Matos, Agostinho Antunes

**Affiliations:** 1 CIIMAR/CIMAR, Interdisciplinary Centre of Marine and Environmental Research, University of Porto, Terminal de Cruzeiros do Porto de Leixões, Av. General Norton de Matos, s/n, 4450-208 Porto, Portugal; anabastosmatos@gmail.com; 2 Department of Biology, Faculty of Sciences, University of Porto, Rua do Campo Alegre, s/n, 4169-007 Porto, Portugal

**Keywords:** ascidians, symbiosis, bioactive potential

## Abstract

Associations between different organisms have been extensively described in terrestrial and marine environments. These associations are involved in roles as diverse as nutrient exchanges, shelter or adaptation to adverse conditions. Ascidians are widely dispersed marine invertebrates associated to invasive behaviours. Studying their microbiomes has interested the scientific community, mainly due to its potential for bioactive compounds production—e.g., ET-73 (trabectedin, Yondelis), an anticancer drug. However, these symbiotic interactions embrace several environmental and biological functions with high ecological relevance, inspiring diverse biotechnological applications. We thoroughly reviewed microbiome studies (microscopic to metagenomic approaches) of around 171 hosts, worldwide dispersed, occurring at different domains of life (Archaea, Bacteria, Eukarya), to illuminate the functions and bioactive potential of associated organisms in ascidians. Associations with Bacteria are the most prevalent, namely with Cyanobacteria, Proteobacteria, Bacteroidetes, Actinobacteria and Planctomycetes phyla. The microbiomes of ascidians belonging to Aplousobranchia order have been the most studied. The integration of worldwide studies characterizing ascidians’ microbiome composition revealed several functions including UV protection, bioaccumulation of heavy metals and defense against fouling or predators through production of natural products, chemical signals or competition. The critical assessment and characterization of these communities is extremely valuable to comprehend their biological/ecological role and biotechnological potential.

## 1. Introduction

The productivity and well-functioning of planet Earth are sustained by the multiple interactions among all ecosystems. Complex biotic and abiotic interactions occur in marine habitats supported by a huge diversity and abundance of organisms, with around 226,000 eukaryotic marine species [1]. Among them are Tunicates, a well-represented group of filter-feeding marine invertebrate organisms whose body is covered by an exoskeletal “tunic” composed of cellulose-like polysaccharide, from which derives the term *tunicate* [2,3]. Along with Cephalochordates and Vertebrates they constitute the phylum Chordata [4]. The subphylum Tunicata is divided into three classes: Thaliacea, Appendicularia and Ascidiacea [5].

Ascidiacea is the most diverse and studied class of Tunicates, with approximately 3000 described species [6,7]. Concerning their tunic organization and shared structures, ascidians can be categorized in solitary and colonial individuals. Solitary species possess their own tunic and structures while colonial organisms are usually divided in either compound or social species. Individual organisms that form communities by attaching their bases to each other, without sharing other physiological structures, are designated as social species. On the other hand, compound species are composed by numerous individuals (each one designated as zooid) sharing a common tunic and a common exhalant siphon through which the filtered seawater is expelled [8,9]. Colonial ascidians have both sexual and asexual reproductive forms whereas the solitary organisms have only a sexual life cycle [10].

According to its adult branchial sac structure, Ascidiacea is divided into three orders: Aplousobranchia (colonial organisms), Phlebobranchia and Stolidobranchia (both orders with solitary and colonial forms) [6,11,12].

Despite its sessile adult form, ascidians have a wide geographical distribution, inhabiting polar, tropical and temperate environments, either in shallow or deeper habitats [6,13]. Coupled with this global distribution, they are associated with an invasive potential, affecting native communities and subsequently leading to economic and environmental impacts [3,6].

Besides its ecological importance, ascidian studies have reached considerable interest in the scientific community, particularly for the study of evolutionary processes due to their close phylogenetic association to vertebrates [4,14,15,16,17,18]. Moreover, the discovery of secondary bioactive metabolites, important to pharmaceutical and biotechnological applications, also contributes to the growing interest in these marine invertebrates [19]. The isolation of bioactive compounds with several distinct activities (e.g., antibacterial, anti-inflammatory) has been described [20,21]. Some of these metabolites have been applied in cancer treatments, such as the case of ET-743 (Yondelis^®^) and Plitidepsin (Aplidin^®^), isolated from *Ecteinascidia turbinata* and *Aplidium albicans*, respectively [22,23,24]. Recently, plitidepsin has gained a renewed attention due to its demonstrated anti-viral activity against SARS-CoV-2 infection [25].

Associations between different species are documented within all domains of life. In 1879, Anton de Bary defined different species living together as “symbiosis” [26]. It can be obligate, facultative or casual [27] and according to the benefits and disadvantages to the involved organisms several concepts are applied (i.e., commensalism, mutualism, parasitism) [27,28]. Later, the concept “holobiont” came up as a reference to a host and its associated community of microorganisms [29]. Lynn Margulis has been credited as the person who firstly introduced the term “holobiont” in 1991, but in fact this concept was introduced years before (in 1943) by Adolf Meyer-Abich [30,31]. Studies aiming to understand and characterize those associations in marine invertebrate organisms are increasing with several worldwide initiatives, as the Global Sponge Microbiome Project [32,33,34,35,36]. The discovery of bioactive compounds, resultant from these associations, and their influence in marine interactions has boosted the studies in parallel with the emergence of the blue economy field. While initially thought to be produced exclusively by ascidians, some of those bioactive compounds are, in fact, produced by their associated organisms [22,37,38,39]. Ascidians’ tunic is a key organ to the protection, development and life cycle of ascidians but also carries out an important role in the establishment of symbioses. This structure enfolds the ascidian body and it is not in contact with digestive and filtration systems [40]. Symbioses in ascidians have been studied, not so extensively, in other organs such as gonads [40,41], gut [42,43] and pharynges [28,44,45].

Hence, with this review, we aim to critically assess the functions and bioactive potential described to date (end of 2020) for the associated organisms of ascidians and demonstrate the benefits that those associations may provide to the involved organisms. This review also provides a brief overview about the performance of the methods applied in the description and characterization of this associated community.

## 2. Bibliographic Research—Selected Criteria

This review was elaborated considering several eligibility and selection criteria. This included information retrieved from studies published until the end of 2020 year (i.e., 31 December) and only when the authors properly identified the ascidian species on which the microbiome study had been conducted. For the purpose of this review, symbiont taxa were defined as organisms living in mutualism and commensalism associations with ascidians. A wide range of studies were referred, from microscopic to metagenomic approaches, focusing on any ascidian’s associations documented so far, with the exception of parasitism. The literature search was conducted accessing PubMed Central^®^, ScienceDirect^®^ and Web of Science platforms. The search was made through a combination of the keywords: “ascidians” and “tunicate” with “microbiome”, “association”, “symbiotic”, “symbiosis”, “isolated”, “-derived”, “microbiota” and “microorganisms”. Ascidians scientific names and status (i.e., accepted, non-accepted name) were confirmed on the World Register of Marine Species (WORMS) database [7].

## 3. Ascidians-Associated Organisms—Knowledge of Their Ecological and Biotechnological Roles

**Figure 1:**
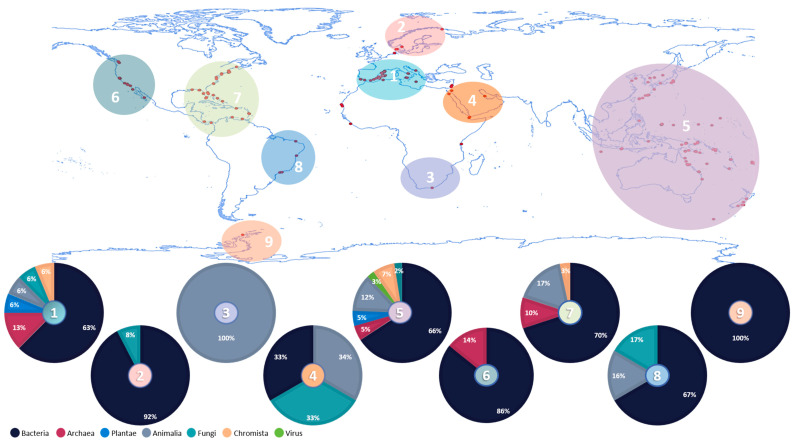
Global distribution of ascidian microbiome studies. The pie charts represent the relative occurrence of each taxon (in percentage) in the different geographical areas where ascidians have been characterized at the microbiome level.

Ascidians are known to establish associations with prokaryotic and eukaryotic organisms (Figure 1). In the following sub-sections, we provide a general overview of the diverse associations documented so far, how they occur, what are the suggested functions of the involved organisms and their bioactive potential. To have a full understanding about the number and type of microbiome studies previously conducted on ascidians, throughout several geographic areas, we compiled detailed information on 171 hosts (Table S1).

### 3.1. Overview of Ascidians’ Microbiome Studies

From the three ascidian orders, Aplousobranchia microbiomes have been the most studied (63%). Phlebobranchia and Stolidobranchia orders presented a similar number of studies, 15% and 22%, respectively. Didemnidae, Ascidiidae and Polyclinidae are the most studied ascidian families and *Didemnum*, *Diplosoma*, *Lissoclinum*, *Aplidium*, *Ascidia* and *Trididemnum* are the most studied genera. In a similar way, within Ascidiacea Class, the highest number of reported natural products belongs also to Aplousobranchia order, in particular to Didemnidae and Polyclinidae families and to *Lissoclinum*, *Didemnum* and *Aplidium* genera [46]. This may explain the interest and the number of microbiomes’ studies focused on these hosts (i.e., Aplousobranchia).

**Figure 2:**
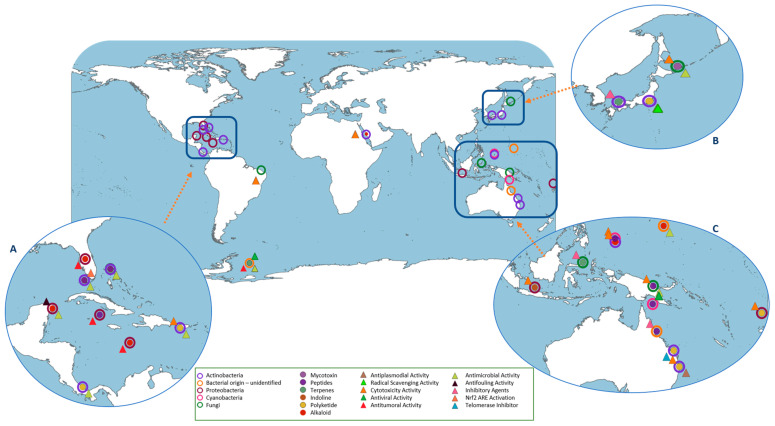
Geographical representation of the sampling points from which the compound producer organisms, associated with ascidians, were described. In the different panels (**A**–**C**), within each sampling point, the corresponding taxa (circles), type of compound (full circles) and compound activity (triangles) are represented.

Globally, the Mediterranean Sea, west Pacific Ocean and Atlantic Ocean (Central America) are the predominant sampling points where the ascidians used for symbiotic studies were collected (Figure 1). Besides the association with natural compounds production, the number of microbiome studies with species collected at those sampling sites may be related with previous description of ascidians in those areas. Consequently, a significantly higher diversity of associated organisms is described in those points due to the higher number of conducted studies. In total, from our review, around 50 phyla from the different domains of life (Archaea, Bacteria, Eukarya) have been detected in association with ascidians, revealing an extraordinary diversity of associated organisms. Regardless of the geographical area, the microbiome structure is suggested to be dominated by bacteria, but associations with other organisms have also been reported (Figure 1). Nevertheless, it should be kept in mind that this high predominance of associated bacteria in association with ascidians compared with other taxa could be biased by the applied approaches and study designs, underestimating the presence of other organisms (see Section 7). The microbiome community is suggested to be the true producer of some of ascidians’ secondary metabolites. In this regard, we also collected information about compounds from which microbial origin has been confirmed (i.e., Fungi, Bacteria) or suggested. Several types of compounds (i.e., terpenes, alkaloids, polyketides) with a wide range of activities (i.e., antibacterial, antitumoral, cytotoxicity) were compiled and their first report geographically represented in Figure 2 [38,39,47,48,49].

As observed in the geographic distribution of the reported ascidians’ microbiome studies (Figure 1), the geographical areas from which the compounds producers were isolated are mainly attributed to Indo-Pacific and Central America (Figure 2).

### 3.2. Prokaryotic Associations

#### 3.2.1. Bacteria

Bacteria occur in all environments as free-living microorganisms but also in association with other organisms, having important functions in ocean biogeochemical cycles [50]. Their presence is well documented in the ascidians’ microbiome, being the best represented group with around 29 phyla reported from which we will highlight the five most represented (Table S2). Among them, Proteobacteria stand out as the most reported phylum in ascidians’ microbiome studies.

Indeed, considering our analysis, Proteobacteria have been detected in 53% of all the considered ascidians (Table S1), with the highest diversity in terms of associated genera (305) (Table S2). Alphaproteobacteria and Gammaproteobacteria are the most reported classes on ascidians’ microbiome studies [13,28,40,51,52,53,54,55], described in 39% and 34% of the analysed hosts, respectively. Although the ecological roles attributed to these associated organisms are yet to be fully determined, some of them have been disclosed. *Endozoicomonas*, is a Gammaproteobacterium genus extensively reported in marine invertebrate microbiomes, whose three main functions were summarized by Jessen and collaborators: nutritional, host health or disease and structural roles in the host microbiome [56]. Its role in ascidians defence against bacterial infections and predators has been already documented [28]. On the other hand, the detection of these bacteria in *Ciona intestinalis* gut suggests their involvement in the metabolism of organic compounds and in sulphur cycling [42,57,58]. Besides, ascidians mucus layer that covers the pharynx is suggested as an important nutritional source for these bacteria [28]. *Endozoicomonas* have also been described in the microbiomes of corals and sponges. Signalling molecules associated with quorum-sensing, such as N-acylhomoserine lactones, and antimicrobial compounds are produced by *Endozoicomonas* and they have been suggested to have structural effects on these organisms’ microbiomes [56,58,59,60]. Quorum-sensing is a mechanism of cell-cell communication where autoinducers are released and the changes in these metabolite concentrations are detected by bacteria. These bacteria then modify their gene expression and consequently their behaviour could be synchronized to function as a multicellular organism [61]. In a similar way, this mechanism may occur in ascidians’ microbiome. The genus *Endozoicomonas* has been detected in several ascidian hosts such as *Ascidia*, *Ascidiella*, *Botryllus*, *Ciona*, *Didemnum*, *Eudistoma* and *Styela* (Table S1) [28,42,62,63,64,65].

*Pseudoalteromonas* is also a Gammaproteobacterium genus commonly described in ascidians’ microbiome, apart from other hosts and marine samples, and associated to the production of secondary metabolites [66,67]. Within *Pseudoalteromonas*, a special attention has been given to *Pseudoalteromonas tunicata*. This bacterium was firstly isolated from *C. intestinalis* and exhibits antibacterial and antifouling bioactivities [68,69,70]. In a study comparing *Pseudoalteromonas* species in diverse samples (marine and eukaryotic hosts), *P. tunicata* in conjunction with *Pseudoalteromonas ulvae* showed the highest level of antifouling activity [67]. A reduction in the settlement of fouling organisms was associated with this activity, and suggested to be the cause of the lowest diversity observed in *C. intestinalis* surface [67]. The ability of ascidians to synthetase cellulose could have triggered the establishment of this association since members of *Pseudoalteromonas* are known to degrade polysaccharides. In this way, *P. tunicata* may use this mechanism as a food source or as a specific surface substratum for its attachment [67]. The inhibitory fungal colonization by *P. tunicata* and the production of an antibacterial protein (AlpP) suggest a competitive advantage (obtention of nutrients and space) when colonizing substrates in the presence of other bacteria [71,72]. However, ascidians might also have an important role in the establishment of this association due to the presence of cellulose in their tunics. Their cellulose fibers are suggested to increase both the attachment of the bacterium to the host surface, by inducing the formation of pili, and the expression of antifouling compounds since this is associated with the same gene that regulates the expression of pili [67].

Another Gammaproteobacterium species related to antifouling activity commonly found in ascidians’ microbiome is *Acinetobacter* sp. (Table S1). When isolated from the ascidian *Stomozoa murrayi* (now accepted as *Stomozoa roseola*), the reduced fouling detected in this host was associated to the 6-bromoindole-3-carbaldehyde compound produced by *Acinetobacter* (Figure 2B) [73]. A metabolite with such bioactivity can act as a defensive strategy of ascidians against predators, as previously suggested for similar compounds [74]. Within Gammaproteobacteria-ascidian associations, some genera have been reported as the main responsible of compound production. One of them is ET-743 (Yondelis^®^), an approved anti-cancer drug, isolated from the ascidian *E. turbinata* [48], produced by *Candidatus Endoecteinascidia frumentensis* (Figure 2B) [22]. The complete assessment of Ca. E. frumentensis genome suggested that ET-743 is a crucial metabolite in this ascidian-bacterium interaction [75]. This hypothesis is based on the preservation of the ET-743 gene cluster in its highly reduced genome, likely the only remaining natural product gene cluster present within this genome [75]. The dispersion of ET-743 biosynthetic genes throughout the genome indicates *Ca. E. frumentensis* as a specialized bacterium in the production of ET-743 metabolite [75]. Previously, reports of the unpalatable characteristic of larvae of *E. turbinata* were described [76] leading Schofield et al. (2015) to suggest that the secondary metabolite ET-743 could function as this deterrent agent in a defensive mechanism avoiding the predation of ascidians [75].

An additional secondary metabolite is Palmerolide A (PalA), with cytotoxicity against melanoma, isolated from the colonial ascidian *Synoicum adareanum* [77]. The levels of PalA in *S. adareanum* were abundant and ubiquitous, along with a core and stable microbiome [65]. Since this compound resembles in structure a hybrid non-ribosomal peptide-polyketide, with similarities to microbially-produced macrolides, it is likely of bacterial origin [65]. *Microbulbifer* is one of the genera suggested as its producer [65]. This genus has been commonly found in ascidians’ microbiome (Table S1), and in sponges microbiome studies is associated with the production of compounds (parabens) [78]. In ascidians, the ecological roles of the production of such compounds are yet to be determined. However, *Microbulbifer* has cellulolytic organisms, which may explain their presence on ascidians tunic, raising questions about the nature of this interaction [79].

Another interesting group of microorganisms are the aerobic anoxygenic phototrophic (AAPs) bacteria, known to be widely dispersed in the marine euphotic zone, but also detected in freshwater systems [80]. AAPs are suggested to be important in the oceans’ carbon cycle [80], being represented in groups as Alphaproteobacteria, Gammaproteobacteria and Betaproteobacteria [81,82,83]. The expression of a gene associated to aerobic anoxygenic photosynthesis, *pufM*, in ascidians tunic tissues has been already reported [84]. AAP organisms, affiliated to Alphaproteobacteria, such as *Roseobacter* genus, have been detected in ascidians’ microbiome (Table S1). *Roseobacter* has important functions in biogeochemical cycles [85,86] and several properties including aerobic anoxygenic photosynthesis, quorum sensing, reduction of trace metals and secondary metabolites production are attributed to this genus [87,88]. Its association with dimethylsulfoniopropionate (DMSP) degradation has been reported. In fact, when this compound is highly concentrated, these bacteria dominate bacterioplankton communities [85,87]. The intake of DMSP is suggested to be associated with the antimicrobial compounds production [89]. Symbiotic associations between *Roseobacter* and corals have been documented and seem to have a crucial role in the health of these hosts [57]. *Roseobacter* has also been identified in the microbiome of several ascidians’ species, such as *Aplidium conicum*, *Ascidiella scabra*, *Botryllus schlosseri*, *Cystodytes dellechiajei*, *Diazona violacea* and *Pseudodistoma cyrnusense* (Table S1).

A further example is the association between ascidians and Rhizobiales (an order within Alphaproteobacteria) (Table S1) [13]. Rhizobiales are associated to nitrogen-fixation functions, recently described in coral microbiome [90,91,92]. The presence of these nitrogen fixing bacteria suggests a possible role of these organisms in the nitrogen cycles of the ascidians holobiont [13]. To better comprehend the bacterium-host interaction, Danish-Daniel and collaborators sequenced the whole genome of the Alphaproteobacterium *Nitratireductor basaltis* strain UMTGB225 isolated from the ascidian *Didemnum molle* [93]. Genes associated to the reduction of nitrate to nitrite were found when analysing the genome of *N. basaltis* [93]. This nitrification process enables the use of nitrogenous compounds as source of energy originated from the metabolic waste produced by the host which is hypothesized to occur in this species [93].

Species belonging to *Pseudovibrio* (Alphaproteobacteria) genus are widely described in marine invertebrate’s microbiome and ascidians are no exception (Table S1). This genus has also earned attention due to their association with the production of bioactive compounds [94]. Comparative genomics studies with *Pseudovibrio* strains isolated from different sources (seawater and eukaryotic hosts) have been developed [95,96]. In those analysis, *Pseudovibrio* strains did not cluster according to the isolation source, showing their capacity to inhabit different habitats [95]. The ascidian-isolated *Pseudovibrio* revealed more copies of cold-shock protein A coding genes when comparing with the other *Pseudovibrio* genomes [95]. This strain was isolated from an Antarctic ascidian, *S. adareanum*, suggesting a capacity to live in those conditions [95]. Moreover, an ability to degrade glycoproteins/glycoconjugates in the ascidian cell wall matrix is shown by the presence of glycoside hydrolase family 109 with an α-N-acetylgalactosaminidase activity [95]. α-N-acetylgalactosaminidase has been involved in alternative mucin degradation pathway and its presence in the genome of the ascidian-isolated *Pseudovibrio* suggests that this bacterium has a capacity to use the mucous secretion of the tunicate [95].

In addition, the colonial ascidian *Lissoclinum patella* is known to contain patellazoles (cytotoxic polyketides) which were found to be produced by its intracellular symbiont, the Alphaproteobacterium *Candidatus Endolissoclinum faulkneri* [97,98]. These highly toxic compounds are assumed to be involved in the chemical defence of *L. patella* [97].

Along with Proteobacteria, one of the most reported phyla in ascidians’ microbiome studies is Cyanobacteria (present in 52% of the considered hosts) (Table S1) [49,51,52,53,63,99]. However, studies on Cyanobacteria-ascidians’ interactions have been mainly focused on *Prochloron* associations, accounting with 31 different genera documented so far in the ascidians’ microbiome (Tables S1 and S2).

*Prochloron* spp. have been the most reported cyanobacteria in ascidians’ microbiome, living in an obligate symbiosis with several ascidians of Didemnidae family (Table S1) [100,101,102,103]. A well-established and described symbiotic association occurs between *Prochloron didemni* and the ascidian *L. patella* [52,101,103,104,105]. *P. didemni* is essential for both primary and secondary metabolism and survival of their hosts [102]. This cyanobacterium transfers fixed carbon to its hosts and uses their excretion products as its primary nitrogen source. This might be related with the survival of ascidians in habitats with low nutrients and/or environments with high luminosity [101,103,106]. On the other hand, ascidians tunic provide protection against ultraviolet (UV) radiation by presenting mycosporine-like amino acids (MAAs) [101,107]. These substances absorb UV but not photosynthetically active radiation, thus promoting the establishment of photosynthetic organisms [101,107]. The photosymbionts are likely the main source of these substances given the high similarity presented with MAAs found in cyanobacterial cells and the presence of MAA biosynthetic genes in cyanobacteria genomes, as demonstrated for *P. didemni* [52,108]. Moreover, toxic secondary metabolites as cyanobactins are produced [52,102,108,109]. Cyanobactin-coding genes are absent in the genome of *P. didemni*’s hosts, being exclusive to its symbiont [110,111]. Patellamides A and C are cytotoxic peptides produced by *P. didemni* and belong to this cyanobactin family [109]. These compounds may act as deterrent agents, aiding ascidians in avoiding predation [100,109]. Some authors consider this an obligate symbiosis [105], but this is still a matter of debate. Unlike obligate symbionts, *P. didemni* genome presents little modifications, no signs of genome reduction, full set of primary metabolic genes and a high G + C content [52]. Within this phylum resides an enormous diversity of strains producing a wide variety of natural compounds with antibacterial or antifouling activities [110].

Actinobacteria phylum is commonly found in marine invertebrate’s microbiome. Ascidians are not an exception being present in 33% of the analysed hosts [13,28,49,53,55,62,63,64,79,112,113,114,115]. However, the exact functions carried out by Actinobacteria in these associations are poorly studied. *Styela plicata*, *C. intestinalis*, *Ascidia sydneiensis samea* and *Ascidia ahodori* are among the hosts associated with a vast diversity of Actinobacteria organisms (Table S1). In ascidians’ microbiomes, the most represented orders of Actinobacteria are Actinomycetales and Acidimicrobiales, and Micromonosporaceae is one of the most reported families (Table S1).

For instance, *Gordonia* is an actinomycete genus associated with ecological roles as bioremediation and biodegradation of pollutants [112,116], which may confer an advantage to ascidians proliferation and survival in adverse environments. Moreover, other potential roles might be associated to the production of bioactive compounds serving as a protective mechanism to ascidians [39]. Within the Micromonosporaceae family, several bioactive secondary metabolites have been identified. Among others, a potent antibiotic, arenimycin, with antimicrobial activity against rifampin- and methicillin-resistant *Staphylococcus aureus*, was isolated from *Salinispora arenicola*, living in association with the ascidian *E. turbinata* [117]. Moreover, in *Salinispora pacifica* the gene cluster responsible for the biosynthesis of lomaiviticin, an antitumor antibiotic, was identified. This compound had been previously isolated from the ascidian *Polysyncraton lithostrotum* without knowing its true producer [118]. Furthermore, it was suggested that staurosporine, isolated from the ascidian *Eudistoma toealensis*, is produced by the actinobacteria *Salinispora* and *Verrucosispora*, helping the ascidian to defend from predation due to its cytotoxicity [119]. From a *Nocardia* strain, isolated from *Trididemnum orbiculatum*, new compounds with antibacterial activity were detected. As an example, peptidolipins were isolated from the abovementioned actinobacterium revealing antibacterial activity against methicillin-resistant and methicillin-sensitive *S. aureus* [120]. *Streptomyces* strains are one of the most prolific sources of natural compounds in Actinobacteria. The analysis of *Streptomyces* genome has led the discovery of a high number of secondary metabolite biosynthetic gene clusters, corroborating the potential to produce secondary metabolites [121].

Moreover, Planctomycetes and Bacteroidetes are two other phyla commonly reported in ascidians’ microbiome. Planctomycetes are described in 24% of the considered hosts, comprising 8 different genera. Their highest diversity detected in *Ascidia ahodori*, *Ascidia sydneiensis samea* and *Styela plicata* (Tables S1 and S2). Comparing with other invertebrate hosts, ascidians exhibit a higher diversity of Planctomycetes [13,122]. Pirellulaceae and Planctomycetaceae have been the most documented families of planctomycetes associated with ascidians (Table S1). Besides other ecological roles, this phylum has been associated with anaerobic ammonium oxidation (Anammox) and recently with nitrogen fixation [123,124]. However, in ascidians its role is still not clear. On the other hand, Bacteroidetes have 109 different genera described in 34% of the hosts and their highest diversity is documented in *Ascidia ahodori*, *Ascidia sydneiensis samea*, *C. intestinalis*, *Eudistoma toealensis*, *Styela plicata* and *Synoicum adareanum* microbiomes (Table S1). One of the most reported Bacteroidetes class in ascidians’ microbiome is Flavobacteria (Table S1). This class is associated with pathogenicity in several organisms, such as fish and even humans [125,126]. This pathogenicity is related with the production of polymer-degrading enzymes, affecting host cellular components [125]. However, in jellyfish, a role in nutrition (pre-digestion of the prey) was also documented [127]. In ascidians’ microbiome, the role of Bacteroidetes is not clear.

#### 3.2.2. Archaea

Another domain frequently detected in association with ascidians is Archaea. The presence of three archaea phyla, Thaumarchaeota, Euryarchaeota and Crenarchaeota, has been reported in ascidians’ microbiome (Table S1) [13,51,64]. Archaea have extremely important roles in biogeochemical cycles [128]. In our review, the highest diversity of archaea was detected in *L. patella* microbiome (Table S1). Moreover, the most reported archaea was *Nitrosopumilus*, a Thaumarchaeota genus associated with ammonia-oxidizing and detoxification processes in sponges tissues, suggested to happen in ascidians as well (see Section 4) [13,51,129,130]. This genus has been described as part of diverse ascidian symbiotic communities, such as *C. dellechiajei*, *Distaplia bermudensis*, *Eudistoma toealensis*, *Herdmania momus*, *Polyandrocarpa anguinea*, *Polyandrocarpa zorritensis* and *Styela plicata* (Table S1) [13,40,51,128,131].

To sum up, although prokaryotic organisms are well reported in ascidians’ microbiome, their actual functions are not clearly understood. However, there are already some clues about the functions (e.g., defence, antifouling activity) developed by some members, essentially Proteobacteria, which highlights the importance of further studies. In comparison with Bacteria, Archaea has been less described in ascidians’ microbiome (Figure 1, Table S2). Nevertheless, the presence of Archaea might be underestimated since the molecular and cultivation approaches applied to microbiomes studies are associated with some bias, as described in Section 7.

### 3.3. Eukaryotic Associations

#### 3.3.1. Fungi

Fungi are extremely important in energy and nutrient regeneration cycles being also part of ascidian symbiotic communities [132,133]. Ascidians tunic is suggested to confer a favourable and stable environment to fungi growth and long-term storage of its spores [134]. The presence of this group of organisms in ascidians as well as in sponges and algae microbiomes was extensively studied in Brazil [132]. Menezes and colleagues found the highest diversity of filamentous fungi in the ascidian *Didemnum* sp., among a sample of 8 marine invertebrates and 1 algae [132]. The fungi species detected are found both in marine and terrestrial environments. Ascomycota was the predominant phylum; these organisms are known to be widely dispersed in the aquatic environments and associated to marine invertebrates. However, this dominance was suggested as a consequence of their easy cultivation and frequent recovery in laboratory conditions [132].

From our analysis, the highest diversity of fungi has been detected within *Didemnum* genus (Table S1). The most described fungi members associated with ascidians belong to Ascomycota. Within this phylum, some strains are source of bioactive compounds [132,133], for instance, trichodermamides A and B, isolated from *Trichoderma virens* associated with the ascidian *Didemnum molle* (Figure 2C). Trichodermamide B exhibited cytotoxicity against HCT-116 human colon carcinoma and antimicrobial activity against *Candida albicans*, *S. aureus*, and *Enteroccus faecium* [135].

Several compounds of ascidian-derived fungi have been isolated. However, their ecological roles are yet to be fully determined. A few studies have already tried to unravel this association, by evaluating ascidian-derived fungi as promoters of biodegradation processes (i.e., as the degradation of xenobiotics). The ascidian derived-*Penicillium citrinum* and *Fusarium proliferatum* proved to be promising strains in the biodegradation of a pesticide (MP), degrading the main toxic metabolite (PNP) [136]. The biodegradation of another pesticide (PCP) by fungi strains isolated from *Didemnun ligulum* was also assessed. *Trichoderma harzianum* was shown to metabolize PCP as well as biodegrade PCA and 2,3,4,6-TeCA metabolites [137]. Apart from their biodegradation capacity, the bioactivity of compounds from fungi strains isolated from ascidians, has also been tested and their antifungal and antibacterial activities demonstrated [138,139,140].

#### 3.3.2. Apicomplexa

The Apicomplexa phylum is well known for having parasite organisms, as is the case of *Plasmodium falciparum*, the Malaria-causing agent. In ascidians, the presence of *Nephromyces* organisms is described in Molgulidae family [141] and *Cardiosporidium cionae* is detected in *C. intestinalis* [142]. However, in this sub-section, a special attention will be given to *Nephromyces* since a mutualistic association between *Nephromyces* and their hosts, instead of parasitism, has been suggested [143]. Contrasting with a parasitic relationship, the mutualistic association is supported by the fact that *Nephromyces* has a higher abundance prevalence in their hosts with 100% infection rate [143]. In their hosts, *Nephromyces* is found in the renal sac, exclusive of Molgulidae ascidians, which contains high levels of uric and oxalic acid deposits [144]. Recently, the uric acid has been suggested to be the primary nitrogen and carbon source for *Nephromyces* [145,146].

Symbiotic associations between *Nephromyces* and Molgulidae ascidian members have been documented and their evolutionary history is suggested to be correlated [143,147]. So far, this symbiotic association has been described in every *Molgula* species and associated to horizontal transmission. However, as mentioned above, in other Molgulidae members as *Bostrichobranchus pilularis* this has also been reported [147].

In general, prokaryotic associations have been most detected in comparison with eukaryotic associations (Figure 1). Nevertheless, the description of eukaryotic members described in association with ascidians, and the metabolites described so far resultant from these associations suggest that interesting and diverse ecological roles of these symbionts may occur and need to be further explored.

## 4. Survival and Proliferation of Ascidians—The Microbiome Role

Ascidians are sessile marine invertebrates associated to an enormous dispersal and invasion capacity. Ascidians’ capacity of dispersion, proliferation, and growth in polluted environments (i.e., marinas and harbours), where the presence of heavy metals at high concentrations, nitrogen and sewage is frequent, is associated with their microbial composition [51,131]. Ammonia-oxidizing organisms are commonly found in ascidians tunic, providing the removal of nitrogenous waste and making them a key player in nitrogen cycle [13,51]. Those symbiotic associations are beneficial for both involved organisms—ammonia-oxidizers process and remove ascidians nitrogenous waste while their growth is supported by the high levels of ammonia present in ascidians tunic [51]. Bacteria involved in nitrogen cycle, as *Nitrospina* (nitrite oxidizer) and *Mesorhizobium* (nitrogen-fixing bacteria), and Archaea, as *Nitrosopumilus* strains (ammonia-oxidizing), are part of ascidians symbiotic community (Table S1) [40,63]. Moreover, the presence of bacterial lineages involved in ammonia (Nitrosomonadaceae family) and nitrite oxidation (phylum Nitrospirae) in the same microbiome leads to the assumption that aerobic nitrification can also occur in ascidian tunics, as in the case of *Eudistoma toealensis* and *Pseudodistoma crucigaster* [64,115]. Denitrifying bacteria, also part of nitrogenous removal processes, are also found in ascidians symbiotic community, as is the case of *Rhodobium orientis*, *Novispirillum* and *Hyphomicrobium* bacterial strains (Table S1) [51,84,131].

In addition, bacterial families associated to heavy metal resistance, such as Hyphomicrobiaceae, Alteromonadaceae or Vibrionaceae, are also present in the ascidians’ microbiome (Table S1). High levels of vanadium are accumulated in blood cells of ascidians belonging to Phlebobranchia order; this metal is absorbed from seawater in a +5 state, then the reduction to a +4 state occurs in ascidians tissues and it is accumulated in a +3 state in vanadocytes (specialized blood cells) [148]. This sequestration capacity has been associated to ascidians bacterial symbionts as *Pseudomonas*, *Ralstonia* and *Shewanella* [148,149].

**Figure 3:**
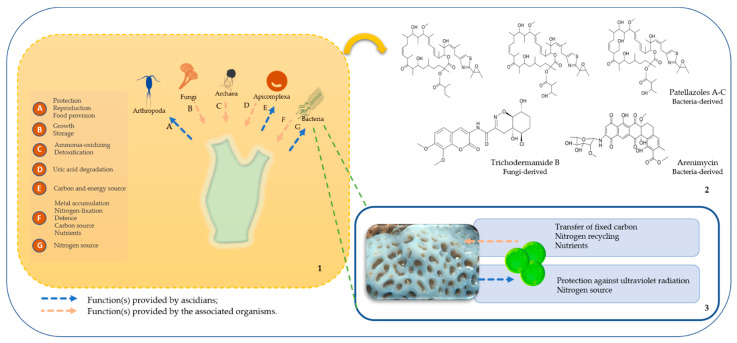
Ascidians’ associated organisms and their biotechnological relevance. In panel 1, the potential roles attributed to each interaction are summarized. In panel 2, compounds derived from ascidians associated organisms are represented. Panel 3 shows the specific association and the roles associated to *Prochloron* and their hosts.4. Survival and proliferation of ascidians—the microbiome role.

A comparative study was performed on samples derived from three ascidian tissues associated to vanadium absorption in two vanadium-rich and one vanadium-poor ascidian species [148]. *Pseudomonas* species (*Pseudomonas brenneri*, *Pseudomonas moraviensis*, *Pseudomonas* sp.) and *Ralstonia* were abundant in branchial sac of vanadium-rich ascidians. These are suggested to be involved in the transition of vanadium to the branchial sac or intestinal lumen. In the intestine, the abundant genera (*Treponema* and *Borrelia*) likely attach to its surface or live within intestinal cells as symbionts, aiding in the accumulation of vanadium from the intestinal lumen. *Treponema* and *Borrelia* genera were also abundant in the intestinal content, which reinforces their involvement in vanadium accumulation [148,149]. *Vibrio* and *Shewanella* are suggested to increase the concentration of vanadium in the intestinal lumen facilitating vanadium sequestration [149]. Another suggested strategy that can aid ascidians colonization is the regulation of their bacterial community for nutritional gains through phagocytosis [53,84,128]. Besides the transfer of nutrients from symbionts to ascidians (e.g., carbon), the extra gathering of nutrients through this mechanism of associated bacteria is suggested to occur in *C. dellechiajei* larvae stages, assisting them in the colonization of new habitats and in sustaining adult colonies [84]. Overall, the associated organisms play several roles in the survival, dispersion, and proliferation of ascidians showing the ecological importance of these interactions (Figure 3).

## 5. Microbiome Diversity Influence on the Metabolome

Within ascidians’ microbiome studies, when comparing the microbiome of different species or strains, a correlation between the presence of a highly diverse microbiome and a higher chemical diversity could potentially be assumed. Some evidence has been shown, as in the case of a research conducted with *C. intestinalis* in which the non-native *C. intestinalis* reported the highest microbial and chemical diversity [150]. However, some authors declare that the production of secondary metabolites is not correlated with a higher microbial diversity but, in fact, to interactions that may occur between some of those secondary-producer organisms that result in more compounds [63]. This was observed by Buedenbender and collaborators, whose microbiome and chemical study of three ascidians species, through culture-dependent and culture-independent methodologies, found the highest chemical diversity in the species with lowest microbiome diversity [49]. A study conducted with *L. patella* showed that, within their three different phylogenetic groups, resided different metabolites [151]. Kwan and collaborators found that the occurrence of host selection capacity by *L. patella* is based on secondary metabolites production, since populations of cyanobactin-producers *P. didemni* relied on host-phylogeny [151]. The presence of natural compounds was also assessed and followed the previous phylogenetic relationship even though their bacterial producers did not correlate with host phylogeny. These authors also showed that, despite the similar genomes of *P. didemni* strains, they produce different compounds, which may suggest a possible selection by the hosts for symbionts based on their secondary compound production (horizontal transmission) [151]. These findings were also corroborated with a posterior study with 32 different ascidians, reinforcing an association between secondary metabolism and host phylogeny [63]. Tianero and collaborators (2015) suggested that in environments with a higher pressure for defensive metabolites production, symbiotic associations are established between ascidians and bacteria capable of producing those compounds [63].

Summarizing, it is important to consider that a high diversity of compounds is not always associated with a high diversity of microorganisms but instead to specific requirements of the host and possible synergistic interactions. Host selection capacity may also influence this, as will be further discussed in Section 6.

## 6. Factors Affecting the Microbiome Composition

Despite the current evidence of the occurrence of host-specific associations (explored in the following paragraphs), the microbiome composition may also mirror the surrounding environment and composition. When comparing seawater with microbiome composition, the occurrence of similar organisms inhabiting these two systems is commonly found. However, particular cases point to the occurrence of enrichment of specific bacteria in the host. This was recently demonstrated by Casso et al., who showed that the 10 most abundant ZOTUs (representing 90% of the reads of the tunic samples) corresponded to only 1.39% of the total reads of seawater samples [152]. The authors relate this with the occurrence of horizontal transmission and with selective enrichment by the hosts [152]. These mechanisms have also been suggested to happen in *Herdmania momus* and *Styela plicata* due to the high degree of intra-species variation found within and between locations [131].

Other factors, such as temperature and light intensity have been reported to play a role in microbiome composition. In a worldwide analysis, Casso and collaborators found evidence of temperature ranges shaping the ascidian *Didemnum vexillum* microbiome [152]. Moreover, a stratification of Cyanobacteria and Proteobacteria was already observed in *L. patella* tunic samples—with the increase of sampling depth, Cyanobacteria became more dominant than Proteobacteria, revealing a possible effect of the environment in microbiome composition [99]. The authors correlate this with ecological parameters such as oxygen levels and light intensity but the occurrence of microenvironments in the ascidians tunic might also contribute to this stratification [99]. The presence of three different microniches in the same tunic tissue, with different associated organisms, was observed [99]. Behrendt et al. studied the microbial communities present in the outside, inside the cloacal cavity and the underside of *L. patella* [99]. In these three different microniches a higher diversity was found in the underside comparing with cloacal cavity and surface samples [99]. Phototrophic bacteria were found in all the three environments whereas chemotrophic bacteria dominated the underside (at intermediate depths) and the surface (at shallow depths), correlated with light and oxygen availability. In the surface of *L. patella*, the presence of high-light environment might select for phototrophic organisms with protective mechanisms, allowing them to photosynthesize under such high-light intensity environments [99]. Regarding oxygen, the more pronounced variation was found in the cloacal cavity (anoxia in darkness to supersaturating conditions during light). The presence of such conditions, as suggested by the authors, presumably contribute to the selection of symbionts with specific adaptations to those conditions [99]. Moreover, acidic conditions present in the tunic of *Didemnum* sp. were suggested to create a selective environment, resulting in the lowest bacterial diversity found on its tunic in comparison with two other colonial ascidians [41]. In contrast, in *C. intestinalis* the variation of microbiomes was more pronounced between individuals than between samples of the same tunic or between tunic and cuticle samples [54]. This study was conducted with species collected on the same sampling site; thus, these findings question if each organism might have its own distinct microbiome [54]. A study conducted by Cahill et al. with four different ascidian species (*Ciona robusta*, *Cionasavignyi*, *Botrylloidesleachi* and *Botryllus schlosseri*), collected in three different sampling sites, also demonstrated that each one of them had a distinct and constant microbiome, with a rare presence of abundant bacteria detected in the seawater [62]. Particular characteristics present in the hosts, mentioned previously, may provide a specific environment for the growth and proliferation of certain microbial communities and contribute to microniches which is supported by the occurrence of host-specificity [13,51,99].

Throughout ascidians’ microbiome studies, host specificity has been suggested to occur when patterns of specific associations between a symbiont and a host are observed. As an example, *Planktothricoides* (Cyanobacteria) appear to inhabit only ascidian tissues [99]. A congruency between phylogenies of *Prochloron* and their hosts was described [105]. *Procholoron* presented more similarity with hosts from the same species located in different and distant geographic origins than with different hosts inhabiting closing located sampling points, suggesting the occurrence of host specificity [105].

The analysis of ascidians’ microbiome collected in tropical, subtropical and temperate environments revealed host-specificity and stability in most of the studied microbiomes [63]. Comparing the microbiome of three different host species collected at the same location and time, Tianero and collaborators found that the major component of the studied microbiomes was host specific, revealing no effect of transmission from seawater or other environmental factors [63]. Besides, no seasonal differences (comparing samples collected in spring and fall of the same year and at the same collection point) were detected [63]. In addition, these findings were also corroborated through the analysis of *L. patella* samples collected in different years and points, which enforced the presence of a host-specific association by the absence of change of their microbiome across time and space [63]. Conservation and stability of microbiomes in ascidians as *C. dellechiajei* and *Didemnum fulgens* (for over a year) has been reported in spite of shifts in seawater bacterioplankton [53,84]. As reported in some species (e.g., *Pseudodistoma crucigaster*), ascidians have resting phases (non-filtering/non-feeding colonies) in their life cycle [115]. A comparative study with these two life forms also demonstrated the presence of a stable core bacterial community [115].

Comparing ascidian microbiomes with the surrounding seawater may also inform and provide hints about a possible specific association occurring in those hosts. A low overlap between the microbial communities found in these two different environments and a strong difference in the relative abundance (by more than an order of magnitude) were detected in a study comparing 42 ascidian species [13]. Around 71% of symbiont OTUs (operational taxonomic units) were only detected in a single host species and not detected in seawater samples, which also indicates the occurrence of host-specific associations [13]. Evans and collaborators also corroborated the hypothesis of host-specific associations, by comparing three ascidian species. Even though the two congeneric species studied demonstrated higher similarity between them, a structural significant difference in their microbiomes was observed [51]. The difference between the OTUs detected in seawater and ascidians’ microbiome was also demonstrated—more than 50% (53.2%) were only detected in ascidians hosts [51].

Nevertheless, several sequences retrieved from ascidians’ microbiomes are phylogenetically similar with sequences retrieved from symbiotic studies targeting other marine invertebrate organisms such as corals and sponges [53,54]. This may suggest that several bacterial lineages are well adapted to symbiotic associations with non-specific hosts, as the case of *Pseudovibrio* (Section 3.2.1) [95,153].

Overall, the composition of the microbiome can be altered by several mechanisms. However, the maintenance of symbiotic associations and/or the establishment of new ones rely on symbiont transmission. Given their importance, these processes are further explored in the following sub-section.

### Symbiont Transmission

Several studies have been focusing on understanding how ascidians acquire their symbiotic community [28,40,51,53,100,154]. Focusing on *Prochloron*, several mechanisms of its transmission in ascidians have been described. The localization of those cyanobacterial cells in the ascidian hosts provides hints about the occurrence of vertical or horizontal transmission strategies [97,100,103,106]. *Prochloron* cells, commonly found in host larvae, are vertically transmitted in ascidians genera as *Didemnum*, *Trididemnum*, *Diplosoma* and *Lissoclinum* [100,103,155,156]. Several adaptations for vertical transmission of *Prochloron* in host larvae have been reported, such as the adherence of cyanobacteria to the tunic of the larval trunk in non-*Diplosoma* species or the attachment of *Prochloron* to a rastrum of the larvae in the case of *Diplosoma* species (reviewed in [100]). The symbiotic association between ascidians and *Prochloron* and their vertical transmission are suggested to have established and evolved independently, in each didemnid genus [155]. According to the distribution patterns of *Prochloron*, as reviewed by Hirose, when these cyanobacteria cells are found on the surface of hosts (e.g., non-didemnid species), a facultative association is suggested [100]. Vertical transmission has never been documented in these cases [100]. However, the analysis of *P. didemni* genomes associated with didemnid ascidians and located at different geographic sampling points revealed a high nucleotide sequence identity among them (>97%), suggesting that they are not genetically isolated and at least a fraction of *P. didemni* may move between hosts [102,151].

Besides *Prochloron*, the presence of other symbionts in organs as gonads or hosts’ larvae, suggests their vertical transmission. The intracellular symbiont *Ca. E. faulkneri*, only found in a subgroup of *L. patella*, is vertically transmitted and this association is suggested to be a case of host restriction [97]. In those cases in which the symbionts are obligate (host restriction) and, consequently, vertically transmitted, changes in the genetic structure of the involved organisms occur [157]. Symbiont transmission methods may also play a role on the survival and proliferation of ascidians.

Vertical transmission methods confer evolutionary and competitive advantages to ascidians since, at the beginning of their development, essential symbionts contribute to the growth and survival of their hosts. However, the vertically transmitted symbiotic community may not be adapted to new colonized habitats [131]. Horizontal transmission (acquisition from the environment) of symbionts helps to overcome the abovementioned factor, since the transmitted symbionts are already adapted to the environment. This process occurs in ascidians, as should be the case of *Styela plicata* due to absence of bacteria in its gonads [40,131]. This type of symbiont transmission is associated to ascidian’ microbiome enrichment when rare seawater bacteria appear to be selectively accumulated in ascidians tunic [28,40,51,131]. Horizontal transmission of symbionts may aid the ascidians’ introduction and invasion in new environments [51]. A recent study comparing native and non-native ascidians species collected at the same sampling site found differences in their microbiome [158]. Intraspecific differences were detected when comparing specimens of the native ascidian *Eudistoma capsulatum* collected in two different sampling sites, harbour and reef systems [158]. On the other hand, for the non-native *D. bermudensis* no significant differences were detected in the same conditions [158]. To some of the associated microorganisms, more than one transmission type is described [152]. These different mechanisms of transmission allow the host to adjust their microbiome composition and maintaining it according to its requirements and the surrounding environment. Therefore, these mechanisms are highly important for ascidian survival and their study is fundamental.

## 7. Approaches Applied in Microbiome Studies

From culture-based and microscopy methodologies to omics approaches, several methods to study ascidians’ microbiome have been described. Initially, culture-dependent and microscopy methods were the main techniques applied. The advent of molecular methods complemented with sequencing approaches revolutionized these studies. They allowed the discovery of new symbionts by detecting uncultured strains, genetic clusters associated with bioactive compound pathways and enabled the use of phylogenetic and phylogeographic analyses. Recently, next generation sequencing (NGS) approaches have been widely applied, producing a massive quantity of data from a single specimen and allowing the discovery of new uncultured symbionts [13,40,51,54,63,64,102,115]. NGS-based approaches provide a higher depth in the analysis of microbial richness compared with the traditional sequencing methods [13,40,51,54,99,115].

However, several biases are implicit in the approaches applied in microbiome’ studies. While these techniques have allowed a breakthrough in the discovery of new associations in ascidians’ microbiome, so far most of those species could not be isolated and cultivated. Moreover, DNA extraction methods, PCR and sequencing protocols, as well as the non–standardization of techniques, present several biases (i.e., amplification efficiency, primer mismatches) that might influence the results obtained and the posterior comparison between studies [159]. Besides the non-detection of uncultivated organisms, in culture-based methods the isolation media are a factor to take in consideration, since they may restrict the growth of certain strains [49].

**Figure 4:**
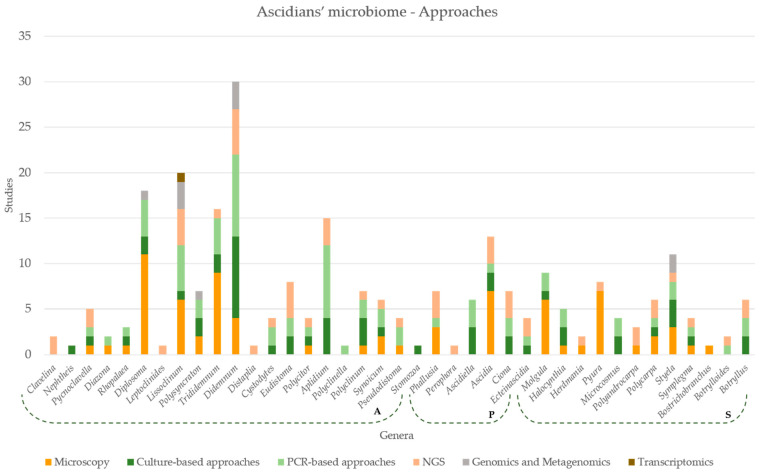
Graphical representation describing the abundance of each approach applied in microbiome studies per ascidian genus. Microscopy comprises light, transmission electron, confocal, fluorescence and scanning electron microscopy. PCR-based approaches comprise Denaturing Gel Gradient Electrophoresis (DGGE), 16S target fragment amplification. NGS comprises pyrosequencing/454 DNA sequencing, Illumina Sequencing. Genomics and Metagenomics comprise sequences extracted from metagenomes and genome sequence of isolated organisms.

Within ascidians’ microbiome field, microscopy (mostly light microscopy) is still the only approach applied in the study of certain hosts (Figure 4). On the other hand, cultivation techniques are systematically associated to PCR-based procedures and, recently, as a complement to NGS techniques (Figure 4). Studies applying integrative and multivariate approaches regarding ascidians’ microbiome have been published in recent years [49]. The application of transcriptomic and proteomic approaches in such studies is still scarce in comparison with the previously mentioned methods (Figure 4) [105,160,161].

However, transcriptomic and metabolomic profiling of the cyanobacteria *Acaryochloris marina* and *Prochloron*, associated to *L. patella*, contributed to a deeper understanding of the regulatory pathways and linkage between these symbionts and host [108]. Chemical diversity of ascidians metabolites has been commonly assessed through liquid chromatography–tandem mass spectrometry (LC-MS) and high-performance liquid chromatography (HPLC) methods [49,63,151,162].

Genome sequencing has been conducted in symbionts isolated from ascidians hosts or retrieved from metagenomic DNA from the host, helping in the assessment of their genetic repertoire and secondary metabolic pathways, as well as in the study of uncultivated organisms [97]. Besides, through metatranscriptomics and metagenomic approaches, the full set of enzymes, metabolic pathways and genes are assessed, elucidating the possible functions of the associated microbiome. However, these approaches are dependent on the availability of reference genomes [163]. Single-cell genomics overcomes this by enabling genome analysis of individual cells giving insights about biotechnological potential and ecological roles of uncultured groups [163].

Application and integration of omics with culture-based approaches complemented by microscopy techniques may lead to a more comprehensive study of microbiomes allowing a better ecological understanding of those associations, assessment of biotechnological potential and to fill the gap about the synergies between host and symbionts that might be responsible for compounds production.

## 8. Conclusions

Worldwide, several studies of ascidians’ microbiome have been conducted. Among them, the most studied hosts so far belong to the Aplousobranchia order, with the main represented genera including *Didemnum*, *Diplosoma*, *Lissoclinum* and *Aplidium*. These studies have revealed a huge diversity of ascidian-associated organisms. The function or the benefit that each association has on both host and symbiont, in general, is not as well understood and documented as the production of metabolites that arise from those associations. Bacteria is the most reported domain, mainly Cyanobacteria, Proteobacteria, Bacteroidetes, Actinobacteria and Planctomycetes phyla, with several functions already attributed, essentially to Proteobacteria phylum. However, the available information regarding the number of ascidians’ microbiome studies and the actual function of the involved organisms is not proportional. Bioactive compounds production, defensive mechanisms and protection/enhancement of capacities against environmental conditions (e.g., UV protection, heavy metal resistance) are within the reported functions of microbiome so far. These symbiotic associations may be dependent on environmental factors. However, each ascidian species may also exert an influence in the establishment of symbioses, adapting its symbiotic community according to its needs. Organisms that can provide several advantageous mechanisms of adaptation are selected to be part of the ascidians’ microbiome. The continuous description and developments on the characterization of ascidians’ microbiome will certainly assist in the characterization of these associations but also in the discovery of new biosynthetic pathways and compounds with strong relevance for biotechnology, biomedicine and pharmacology.

## Data Availability

The data presented in this study are available in supplementary Table S1.

## Supplementary Materials

The following are available online at https://www.mdpi.com/article/10.3390/md19070370/s1, Table S1: Description of each phylum (per host) reported in association with ascidians, Table S2: Occurrence (in percentage) of each phylum in the overall analysis of microbiome studies as well as the number of different genera detected in each of those phyla.
