# Supplementary material for: Symbiotic Associations in Ascidians: Relevance for Functional Innovation and Bioactive Potential
Source: Mar Drugs. 2021 Jun 26;19(7):370. doi: 10.3390/md19070370 (PMC8303170; doi:10.3390/md19070370)
Supplement: Supplementary file 1 [file marinedrugs-19-00370-s001.zip › supplementary - proofread/Table S2.pdf]

Table S2 - Occurrence (in percentage) of each phylum in the overall analysis of microbiome studies as well as the number of different genera detected in each of those phyla.

Phylum	Hosts (%)	Number of Genera
**Proteobacteria**	53	305
**Cyanobacteria**	52	31
**Bacteroidetes**	34	109
**Actinobacteria**	33	95
**Planctomycetes**	24	8
**Chloroflexi**	20	5
**Thaumarchaeota**	20	2
**Arthropoda**	17	12
**Acidobacteria**	17	6
**Firmicutes**	14	120
**Nitrospirae**	12	3
**Ascomycota**	10	33
**Verrucomicrobia**	10	22
**Spirochaetes**	8	4
**Ochrophyta**	7	10
**Fusobacteria**	6	5
**Apicomplexa**	6	3
**Tenericutes**	5	8
**Euryarchaeota**	5	12
**Rhodophyta**	5	4
**Chlamydiae**	5	5
**Chlorophyta**	4	2
**Deinococcus-Thermus**	4	3
**Crenarchaeota**	4	0
**Lentisphaerae**	4	2
**Porifera**	3	6
**Synergistetes**	3	3
**Fibrobacteres**	3	0
**Chlorobi**	3	2

**Deferribacteres**	3	2
**Thermotogae**	2	1
**Armatimonadetes**	2	0
**Nematoda**	2	1
**Gemmatimonadetes**	2	0
**Elusimicrobia**	2	0
**Calditrichaeota**	2	1
**Annelida**	1	2
**Thermodesulfobacteria**	1	1
**Platyhelminthes**	1	1
**Nitrospinae**	1	1
**Cnidaria**	1	5
**Aquificae**	1	0
**Zygomycota**	1	3
**Tracheophyta**	1	1
**Phixviricota**	1	1
**Dictyoglomi**	1	0
**Ciliophora**	1	1
**Choanozoa**	1	1
**Cercozoa**	1	1
**Basidiomycota**	1	2
